# VenoArterial Extracorporeal Membrane Oxygenation in Cardiac Arrest Suspected due to Massive Pulmonary Embolism: A Case Report

**DOI:** 10.5811/cpcem.52963

**Published:** 2026-07-20

**Authors:** Tananshi Chopra, Lea Dahlke, Nancy Salinas, Moizza Shabbir, Tyler Gunn, Sam Torbati

**Affiliations:** Cedars Sinai Medical Center, Emergency Department, Los Angeles, California

**Keywords:** massive pulmonary embolism, cardiac arrest, extracorporeal membrane oxygenation, thrombectomy, resuscitation

## Abstract

**Introduction:**

High-risk pulmonary embolism (PE) is an uncommon but potentially reversible cause of out-of-hospital cardiac arrest, frequently presenting as pulseless electrical activity (PEA). Early identification and multidisciplinary intervention are critical, yet confirmatory imaging is often delayed. Current guidelines recommend consideration of venoarterial extracorporeal membrane oxygenation (VA-ECMO) support in specialized centers when high-risk PE is suspected and the patient presents with cardiogenic shock or cardiac arrest.

**Case Report:**

We describe a 50-year-old woman with a remote history of deep vein thrombosis who arrested after acute-onset dyspnea. Emergency medical services documented PEA arrest and initiated advanced cardiac life support. On arrival to the emergency department (ED), the patient remained in PEA arrest without cardiac motion on point-of-care ultrasound. Pulmonary embolism was strongly suspected but could not be confirmed, and VA-ECMO was initiated within 42 minutes of arrest. Pulmonary angiography confirmed massive bilateral PE, followed by mechanical thrombectomy using a catheter-directed device. Extracorporeal membrane oxygenation support continued for four days before successful decannulation, and the patient was discharged on hospital day 17 to a rehabilitation facility with a Cerebral Performance Category score 1, indicating excellent neurological function.

**Conclusion:**

This case illustrates the potential for favorable neurologic and functional outcomes in cardiac arrest associated with high-risk PE when VA-ECMO is implemented early as a bridge to definitive therapy. In resource-rich settings, rapid ED-based activation of extracorporeal cardiopulmonary resuscitation protocols and access to catheter-directed thrombectomy may represent an optimal strategy for improving survival and neurologic recovery in patients with PEA arrest that may be related to high-risk PE.

## INTRODUCTION

High-risk pulmonary embolism (PE) is an uncommon but potentially reversible cause of out-of-hospital cardiac arrest, frequently presenting as pulseless electrical activity (PEA).^1^ Early identification of pathology and coordination of multidisciplinary interventions are critical, yet confirmatory imaging is often delayed due to ongoing resuscitative measures.^2^,^3^ Current American Heart Association (AHA) and European Society of Cardiology (ESC) guidelines recommend venoarterial extracorporeal membrane oxygenation (VA-ECMO) initiated during cardiac arrest, a strategy commonly referred to as extracorporeal cardiopulmonary resuscitation (ECPR), systemic thrombolysis, and catheter-based thrombectomy in high-resource settings for patients with suspected high-risk PE presenting in cardiogenic shock or cardiac arrest.^2^,^3^ Early clinical decision-making remains challenging for PEA arrest when pulmonary embolism is suspected but not yet confirmed. Conventional CPR is frequently insufficient due to mechanical obstruction of pulmonary circulation and acute right ventricular failure, both of which limit the return of spontaneous circulation.^4^ Advanced resuscitative measures such as VA-ECMO, systemic thrombolysis, and catheter-based interventions have emerged as effective tools to reverse this obstructive physiology in appropriately selected patients.^2^

As the first point of contact for these patients, the emergency department (ED) is uniquely positioned to recognize high-risk PE as a reversible cause of cardiac arrest, initiate high-quality CPR, and rapidly coordinate multidisciplinary interventions. In high-resource settings, emergency clinicians play a critical role in early candidate identification and ECMO activation pathways.^5^ Although a growing body of literature supports the use of VA-ECMO for high-risk PE and the utility of ECPR in refractory cardiac arrest, structured protocols for its use in the ED are still limited.^6^,^7^

We present the case of a previously healthy woman who experienced out-of-hospital cardiac arrest due to high-risk PE and achieved full neurologic recovery via early initiation of VA-ECMO, followed by diagnostic pulmonary angiography and simultaneous catheter-directed thrombectomy. This case demonstrates real-time ED decision-making in alignment with current guideline recommendations for management of high-risk PE in high-resource ED settings.

## CASE REPORT

A 50-year-old female with a remote history of provoked deep vein thrombosis following spinal surgery called emergency medical services (EMS) with complaints of acute dyspnea and leg pain. Paramedics noted that the patient was in severe respiratory distress, tachypneic, and hypoxic with an oxygen saturation of 83% on room air. Supplemental oxygen was provided, followed by initiation of bag-valve mask ventilation for respiratory support. While en route to the ED, she experienced PEA cardiac arrest (Time 0 in Table 1). Chest compressions were immediately initiated, and bag-valve mask ventilation continued during the short transport time.

Upon arrival to the ED at minute 9 post-arrest, the patient was cyanotic, unresponsive (Glasgow Coma Scale 3), and warm to the touch. Recognizing the need for uninterrupted perfusion and rapid diagnosis, the ED team immediately initiated mechanical chest compressions using the Lund University Cardiac Assist System device, established intraosseous access followed by central venous access, performed endotracheal intubation, continued advanced cardiovascular life support medication management, and activated the ECMO team.

*CPC-EM Capsule*

What do we already know about this clinical entity?

*High-risk pulmonary embolism (PE) can cause pulseless electrical activity (PEA) cardiac arrest and carries extremely high mortality despite standard advanced life support.*

What makes this presentation of disease reportable?

*This case demonstrates survival after PEA arrest from high-risk PE using early extracorporeal cardiopulmonary resuscitation (E-CPR).*

What is the major learning point?

*Early recognition of PE as the cause of arrest allows timely E-CPR and definitive reperfusion therapy.*

How might this improve emergency medicine practice?

*In cases of suspected high-risk PE, early extracorporeal membrane oxygenation activation could improve survival.*

Venoarterial extracorporeal membrane oxygenation was initiated with cannulation of the left femoral vein and right femoral artery with 25 and 20 French cannulas, respectively, and the patient was started on the ECMO circuit within 33 minutes of ED arrival (42 minutes post-arrest). Repeat cardiac point-of-care ultrasound performed after 30 minutes of ECMO initiation demonstrated severe right ventricular dilation and biventricular dysfunction, suggestive of high-risk PE. The patient was rapidly transferred from the ED to the cardiac catheterization lab for pulmonary angiography, which confirmed large thrombus burden in the right main and moderate burden in the left main pulmonary arteries. Aspiration thrombectomy with a minimally invasive mechanical thrombectomy device-restored distal perfusion, and an intra-aortic balloon pump was placed to support ongoing cardiogenic shock. The clinical sequence of interventions and physiologic milestones from arrest to ECMO cannulation is summarized in Table 1, and summary of diagnostic findings is provided in Table 2.

Upon admission to the intensive care unit, the patient developed severe upper gastrointestinal bleeding, a recognized complication of cardiac arrest and ECMO-required anticoagulation. She was managed with proton pump inhibitor therapy, transfusions of fresh frozen plasma, cryoprecipitate, and red blood cells. Esophagogastroduodenoscopy demonstrated friable gastric mucosa and bleeding superficial ulcers treated with vessel clipping.

On hospital day 1, transthoracic echocardiography showed a left ventricular ejection fraction of 65% with only mild right ventricular dysfunction. After 100 hours of ECMO support, the patient was successfully decannulated. The intra-aortic balloon pump was removed on hospital day 5. She was extubated on day 6 and discharged on day 17 to an inpatient rehabilitation facility to address residual mild functional and cognitive decline with a Cerebral Performance Category (CPC) 1, indicating an excellent neurological outcome.

## DISCUSSION

This case demonstrates the successful ED-initiation of VA-ECMO for extracorporeal CPR in a woman with out-of-hospital cardiac arrest due to suspected and later confirmed high-risk PE.

Pulseless electrical activity is widely recognized as a non-shockable cardiac arrest rhythm associated with poor survival in numerous recent studies.^8^ In a large retrospective study by Nadkarni et al, only 11.2% of patients with initial PEA survived to hospital discharge, and outcomes were significantly worse than those with initial shockable rhythms such as ventricular fibrillation or tachycardia.^8^ In comparison, cardiac arrest secondary to high-risk PE carries an even higher mortality burden, commonly reported as 95%,^1^ with many patients dying within the first hour of presentation. Another case study reported by Qiu et al also found that mortality in PE-related cardiac arrest ranges from 52–84%, with survival remaining limited even when advanced interventions are used.^6^

Survival after witnessed out-of-hospital cardiac arrest remains dismal.^9^ In a large observational study of over 38,000 EMS-attended cases, survival to hospital discharge was 5.9% for PEA and 1.1% for asystole, with no improvement over a 10-year period.^9^ Among PEA survivors with 12-month follow-up, approximately 45% were either in a vegetative state or had severe disability.^9^ These findings highlight the devastating outcomes typically associated with PEA arrest and show the exceptional nature of our patient’s recovery, who was discharged with CPC 1 neurologic function following her PEA arrest related to high-risk PE.

Venoarterial extracorporeal membrane oxygenation has been shown to improve survival and neurological outcomes in refractory cardiac arrest. A 2024 meta-analysis by Low et al reviewed 13 studies comparing ECPR to conventional CPR and found that ECPR significantly reduced in-hospital mortality while improving 30-day survival along with favorable neurologic outcome (CPC 1–2).^10^ Together, these studies provide robust evidence that early application of VA-ECMO in carefully selected patients significantly improves both survival and neurologic outcomes. This aligns with recent 2019 AHA and ESC guidelines supporting the use of VA-ECMO in patients with suspected or confirmed CPC 1 presenting with cardiac arrest as a bridge to definitive intervention, particularly in centers with appropriate expertise and resources.^1^,^2^

Although systemic thrombolysis remains the first-line therapy for confirmed high-risk PE, especially in cardiac arrest, its clinical utility may be constrained by delays in diagnosis, relative contraindications, or insufficient efficacy in profound obstructive physiology.^1^ Retrospective studies have explored alternative or adjunctive strategies, including ECMO and definitive reperfusion interventions. In a systematic review and meta-analysis by Boey et al, the pooled mortality among patients with high-risk PE treated with ECMO was 42.8%.^12^ Outcomes varied substantially depending on adjunctive therapy: Mortality was highest (57%) in patients treated with ECMO and systemic thrombolysis, while those receiving ECMO combined with catheter-directed thrombectomy had significantly lower mortality at 28.6%.^12^

Comparatively, ECMO alone yielded a mortality rate of 41.3%, and although data are more limited, patients undergoing ECMO with surgical embolectomy demonstrated intermediate outcomes with reported mortality around 37.2%.^12^ In contrast, systemic thrombolysis alone in PE-related cardiac arrest has been associated with a significant increase in return of spontaneous circulation but often without meaningful neurologic recovery.^13^ These findings suggest that in resource-rich centers, a multidisciplinary approach incorporating early ECMO support and catheter-based thrombectomy may offer the most favorable survival and neurologic outcomes in patients with suspected high-risk PE-related cardiac arrest.

Patients surviving cardiac arrest commonly face postcardiac arrest syndrome, characterized by hypoxic‐ischemic brain injury, acute kidney injury, multiorgan dysfunction (acute kidney injury, liver dysfunction, coagulopathy, and shock), arrhythmias, and systemic inflammation.^14^ Patients requiring ECMO are also subject to additional risk beyond postarrest sequelae, notably major bleeding and thrombosis, limb ischemia from femoral cannulation, hemolysis, and infection.^15^ The patient presented in the case report experienced only one major complication—an upper gastrointestinal bleed, which was quickly identified and managed successfully. Her neurologic recovery (CPC 1), early extubation, and hospital discharge by day 17 represent an exceptionally favorable clinical course, particularly given the high morbidity and mortality typically associated with both conventional and ECMO-supported cardiac arrest survivors.

## CONCLUSION

This case demonstrates the successful initiation of VA-ECMO in a woman presenting to the ED setting with out-of-hospital cardiac arrest secondary to high-risk PE. Venoarterial extracorporeal membrane oxygenation in combination with mechanical thrombectomy can provide remarkable patient outcomes in an otherwise high-mortality clinical scenario. The ED plays a crucial role in identifying candidates for extracorporeal CPR and mobilizing multidisciplinary teams to implement time-sensitive interventions. Developing robust ED-driven protocols for early VA-ECMO activation, particularly in arrest associated with high-risk PE, may improve survival and functional outcomes in this otherwise high-mortality population.

## Figures and Tables

**Table 1:** Clinical timeline of events and interventions from arrest to extracorporeal membrane oxygenation initiation in a patient with suspected high-risk pulmonary embolism.

Timeline	Clinical events and interventions
Minute 0 (00:00)	PEA Arrest
Minute 9 (00:09)	ED Arrival
Minute 10 (00:10)	LUCAS/BMV ventilation
Minute 14 (00:14)	IO Access/Orotracheal Intubation
Minute 15 (00:15)	IV Epinephrine 1 mg (#1)
Minute 17 (00:17)	IV Epinephrine 1 mg (#2)
Minute 19 (00:19)	Internal Jugular Central Access Epinephrine infusion (20 mcg/min)
Minute 22 (00:22)	IV Sodium Bicarbonate 50 mEq
Minute 26 (00:26)	IV Epinephrine 1 mg (#3)
Minute 31 (00:31)	IV Epinephrine 1 mg (#4)
Minutes 42 (00:42) *Door to ECMO: 33 min	ECMO flow initialized

Chronological summary of major clinical interventions and physiological milestones from the patient’s out-of-hospital cardiac arrest to the initiation of venoarterial ECMO. Timing is approximate and based on field and emergency department documentation.

*PEA*, pulseless electrical activity; *BVM*, bag-valve-mask; *ECMO*, extracorporeal membrane oxygenation*; ED*, emergency department; *LUCAS*, Lund University Cardiopulmonary Assist System; *IO*, intraosseous; *IV*, intravenous; *mcg*, micrograms; *mEq*, milliequivalents; *mg*, milligram.

**Table 2:** Diagnostic findings and laboratory results upon emergency department arrival and initial resuscitation of a patient with suspected high-risk pulmonary embolism.

	Diagnostic findings and lab results	Reference ranges
POCUS (pre-ECMO)	No cardiac motion	N/A
Troponin (high sensitivity)	35 ng/L	*<*14 ng/L
Lactate	> 20 mmol/L	0.7–2.1 mmol/L
Glucose	342 mg/dL	70–90 mg/dL
K+	5.4 mEq/L	3.5–5 mEq/L
Hgb/Hct	13.8 g/dL/42%	10.3–14.8 g/dL and 32.5–46.2%
Calcium (ionized)	1.15 mmol/L	1.16–1.31 mmol/L
Pulmonary angiography	Large bilateral PE	N/A
ECHO (Hospital Day 1)	EF 65%, mild RV depression	EF 55–70%; RV function qualitative

Key laboratory values and imaging findings that supported the diagnosis of massive pulmonary embolism informed the decision to initiate thrombolysis and ECMO.

*dL, deciliter;* ECHO, echocardiogram; *ECMO*, extracorporeal membrane oxygenation*;* ED, emergency department; *EF*, ejection fraction; *g, gram; Hct*, Hematocrit; *Hgb*, Hemoglobin; *K+*, potassium ionized*; L*, liter; *mEq*, milliequivalents; *mg*, milligrams; *mmol*, millimole; *ng*, nanogram; *PE*, pulmonary embolism; *POCUS*, point-of-care ultrasound; *RV*, right ventricle.
